# Spike Timing Regulation on the Millisecond Scale by Distributed Synaptic Plasticity at the Cerebellum Input Stage: A Simulation Study

**DOI:** 10.3389/fncom.2013.00064

**Published:** 2013-05-22

**Authors:** Jesús A. Garrido, Eduardo Ros, Egidio D’Angelo

**Affiliations:** ^1^Neurophysiology Unit, Department of Brain and Behavioral Sciences, University of PaviaPavia, Italy; ^2^Consorzio Interuniversitario per le Scienze Fisiche della MateriaPavia, Italy; ^3^Department of Computer Architecture and Technology, University of GranadaGranada, Spain; ^4^Brain Connectivity Center, IRCCS Istituto Neurologico Nazionale C. MondinoPavia, Italy

**Keywords:** spiking network, spike timing, cerebellum, granular layer, LTP, LTD

## Abstract

The way long-term synaptic plasticity regulates neuronal spike patterns is not completely understood. This issue is especially relevant for the cerebellum, which is endowed with several forms of long-term synaptic plasticity and has been predicted to operate as a timing and a learning machine. Here we have used a computational model to simulate the impact of multiple distributed synaptic weights in the cerebellar granular-layer network. In response to mossy fiber (MF) bursts, synaptic weights at multiple connections played a crucial role to regulate spike number and positioning in granule cells. The weight at MF to granule cell synapses regulated the delay of the first spike and the weight at MF and parallel fiber to Golgi cell synapses regulated the duration of the time-window during which the first-spike could be emitted. Moreover, the weights of synapses controlling Golgi cell activation regulated the intensity of granule cell inhibition and therefore the number of spikes that could be emitted. First-spike timing was regulated with millisecond precision and the number of spikes ranged from zero to three. Interestingly, different combinations of synaptic weights optimized either first-spike timing precision or spike number, efficiently controlling transmission and filtering properties. These results predict that distributed synaptic plasticity regulates the emission of quasi-digital spike patterns on the millisecond time-scale and allows the cerebellar granular layer to flexibly control burst transmission along the MF pathway.

## Introduction

By operating in a continuously changing environment, neuronal networks have evolved precise processes regulating the number and positioning of spikes (Rieke et al., 1999). Spike timing has been revealed in afferent sensory pathways and in cortical networks (Mackevicius et al., 2012), in which millisecond-scale correlations among neurons are thought to improve information storage capacity and computational capabilities (Petersen et al., 2009; Eldawlatly and Oweiss, 2011; Kimura et al., 2011). Spike timing can be controlled by long-term synaptic plasticity (Nieus et al., 2006), which regulates the strength and dynamic properties of synaptic connections. Nevertheless, it is not clear how differential distribution of synaptic weights could fine-tune spike timing in central networks expressing multiple distributed forms of synaptic plasticity.

**Figure 1:**
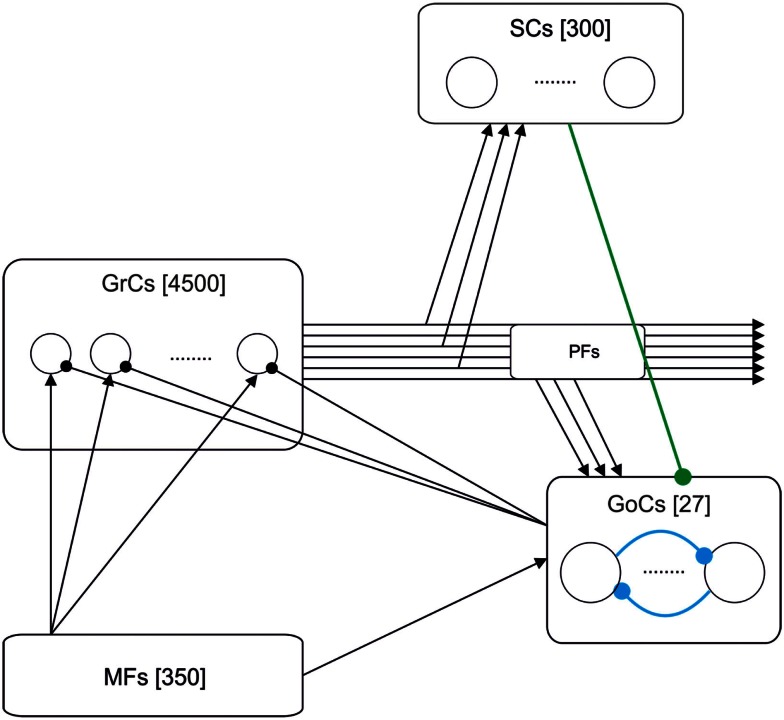
**Schematic drawing of the cerebellar granular-layer model adopted in this work**. The network includes 350 mossy fibers (MFs), 4500 granule cells (GrCs) and parallel fibers (PFs), 300 stellate cells (SCs), and 27 Golgi cells (GoCs). Three different versions of the model have been studied. The basic one (black lines) includes the excitatory pathway (MF-GrC), feed-forward inhibitory loop (MF-GoC-GrC) and the feed-back inhibitory loop (GrC-GoC-GrC). The extended versions add the GrC-SC-GoC-GrC loop (green line) and the GoC-GoC inhibitory connection (blue lines) (Barmack and Yakhnitsa, 2008; Hull and Regehr, 2012).

The cerebellum has long been proposed to operate as a “timing machine” (Eccles, 1967) and a “learning machine” (Ito, 2006), but the intrinsic nature of these operations has not been resolved yet. Interestingly, the cerebellum controls motor behavior with millisecond precision (Timmann et al., 1999; Osborne et al., 2007), so it is expected that its computations are performed on a comparable or even faster time-scale. There are indications, mostly derived from cellular investigations in rat cerebellar slices, that the granular layer (see Figure 1) is capable of exerting a close control on spike timing (D’Angelo and De Zeeuw, 2009). The granule cells (GrCs) generate brief spike bursts in the axon initial segment, which are almost instantaneously (<0.3 ms) transmitted to the dendrites and to synapses on the axonal ascending branch (Diwakar et al., 2009, 2011). Moreover, the mossy fiber (MF) – GrC EPSCs have extremely fast kinetics [rise time <1 ms (Silver et al., 1992)] and can therefore excite the GrCs with high temporal precision (Cathala et al., 2005). Finally, GrCs are endowed with specific ionic mechanisms capable of controlling the delay and persistence of spike emission (D’Angelo et al., 2001). A theoretical analysis has revealed that half of the information carried by MF spike trains is retransmitted by GrCs as first-spike delay with millisecond precision and half as spike frequency (Arleo et al., 2010). Interestingly, long-term potentiation (LTP) and long-term depression (LTD) have been shown to regulate both first-spike delay and spike frequency through different mechanisms (Nieus et al., 2006). The outstanding timing capabilities of this system have been summarized into the “time-window” hypothesis, which considers how these mechanisms compete with feed-forward synaptic inhibition mediated by Golgi cells (GoCs) in order to control spike emission from the GrCs during a period of a few milliseconds after MF burst discharge (D’Angelo and De Zeeuw, 2009; D’Angelo et al., 2013; D’Angelo et al., submitted).

A precise understanding of spike timing in the granular layer needs to consider long-term synaptic plasticity not just at the MF – GrCs synapse but also at other synapses (Gao et al., 2012). Recent results indicate that LTD may indeed occur at the parallel fiber (PF) – GoC synapse (Robberechts et al., 2010), while evidence for long-term synaptic plasticity in the feed-forward MF – GoC – GrCs inhibitory loop is still lacking. However, forms of adaptation may occur at the GoC – GrC synapse (Rossi et al., 2006; Brandalise et al., 2012). The other important element to consider is how network inhibition is organized. Different reports have indicated the presence of stellate cell (SC) – GoC inhibitory synapses (Casado et al., 2000), of GoC – GoC inhibitory synapses (Hull and Regehr, 2012), and of GoC–GoC gap junctions (Vervaeke et al., 2010). This complex connectivity could have an impact on spike timing.

Given the large number of variables, it is desirable that a theoretical and computational analysis is anticipated to predict how a distributed regulation of synaptic weights could control spike timing. To this aim, we have constructed a granular-layer spiking network model accounting for different hypotheses on connectivity in the inhibitory loops and for different assets of weights in circuit synapses. In response to burst stimulation of the MFs, GrCs in the model showed a permissive time-window regulated by inhibition. Variations in the weights at multiple synapses effectively tuned the windows boundaries and the efficiency of inhibition, regulating the delay to first-spike, and the overall number of spikes emitted. Therefore, distributed plasticity can implement an extensive and flexible control over spike timing and bursting on the millisecond time-scale, which could have important implications for cerebellar mechanisms of function.

## Materials and Methods

### Overview of the computational model

A granular-layer model was built using the EDLUT simulator (http://edlut.googlecode.com) (Ros et al., 2006). This model accounts for the topology of connections and neuronal elements included into the realistic granular-layer model developed by Solinas et al. (2010). However, since the aim of this work was to investigate the influence of synaptic weights at multiple connections, neurons were simplified using leaky integrate-and-fire (LIF) models, and synapses were simplified using exponential models (Gerstner and Kistler, 2002). The source code of the model is available at ModelDB (https://senselab.med.yale.edu/modeldb/ShowModel.asp?model=149913).

The differential equations were solved using the fourth order Runge–Kutta method with a fixed time-step of 100 μs. Simulations of 4-s activity trials were carried out on a 152-node cluster located at the *Centro de Investigación en Tecnologías de la Información y las Comunicaciones* (*CITIC*) in the University of Granada.

### The network structure

Figure 1 shows the model of the granular layer including 5177 single-compartment cells. Network topology was developed in two different steps. Initially, the number of constitutive elements was calculated following anatomical studies of cell densities in the granular layer (Korbo et al., 1993). Then, we have connected those elements respecting convergence-divergence ratios and connectivity rules based on biology (Eccles, 1967; Harvey and Napper, 1988, 1991).

The granular-layer model which has been simulated in this work is composed of the following elements:

- Mossy fibers (MFs) (350 fibers): MFs convey the input stimuli and activate GrCs and GoCs.
- Granule cells (GrC) (4500 neurons): The population of GrCs has a number of connections per cell (from MFs) that follows a Gaussian distribution with a mean of four connections and a standard deviation of one connection (Nieus et al., 2006).
- Golgi cells (GoC) (27 neurons): Each GoC receives excitatory connections from 100 GrCs (Nieus et al., 2006) and 50 MFs. The output of each GoC inhibits 667 GrCs (on average). In some simulations, the network included also inhibitory GoC-GoC connections (10:1 convergence).
- Stellate cells (SC) (300 neurons): Each SC receives excitation from 100 GrCs and inhibits the activity of 4.5 GoCs (on average). The SCs have only been included in the model when explicitly stated. Otherwise, the connections between SC and GoC have been disabled.

Since this work is focused on temporal properties, while neglecting specific topologies like the center-surround organization imposed by lateral inhibition [see (Solinas et al., 2010)], all connections have been generated randomly (with the only restriction of avoiding duplication of source-target cell pairs).

### The neuron and synapse models

Neurons were modeled using modified versions of the LIF model (Gerstner and Kistler, 2002; Gerritz et al., 2011). In the LIF model, membrane potential (*V*_m_) is computed through the differential equation (Eq. 1A), which accounts for the effect of chemical synapses [including AMPA, NMDA, and gamma-aminobutyric acid (GABA) receptors] and resting conductance (*G*_rest_),

(1a) $$\begin{array}{llll} C_{\text{m}}\frac{dV_{\text{m}}}{dt} & =g_{\text{AMPA}}\left(t\right)\left(E_{\text{AMPA}}-V_{\text{m}}\right) & & \\ & \;+g_{\text{NMDA}}\left(t\right)g_{\infty ,\text{NMDA}}\left(V_{\text{m}}\right)\left(E_{\text{NMDA}}-V_{\text{m}}\right) & & \\ & \;+g_{\text{GABA}}\left(t\right)\left(E_{\text{GABA}}-V_{\text{m}}\right)+G_{\text{rest}}\left(E_{\text{rest}}-V_{\text{m}}\right) \end{array}$$

(1b) $$\begin{array}{ll} g_{\infty ,\text{NMDA}}\left(V_{\text{m}}\right) & =\frac{1}{1+e^{-\alpha V_{\text{m}}}\left[\text{M}{\text{g}}^{2+}\right]∕\beta } \end{array}$$

Where *C*_m_ denotes the membrane capacitance, *E*_AMPA_, *E*_NMDA_, and *E*_GABA_ are the reversal potentials of each synaptic conductance and *E*_rest_ is the resting potential. The conductances *g*_AMPA_, *g*_NMDA_, and *g*_GABA_ integrate all the contributions received through individual synapses for each of the receptor types (AMPA, NMDA, and GABA, see below). Finally, Eq. 1B determines *g*_∞,NMDA_(*V*_m_), the gating function of the NMDA channels accounting for voltage-dependent magnesium block (Jahr and Stevens, 1990), with α = 62 V^−1^, [Mg^2+^] = 1.2 mM and β = 3.57 mM (adapted from Gabbiani et al., 1994).

The synaptic conductances have been modeled following Eqs 2A–C:

(2a) $$\begin{array}{ll} g_{\text{AMPA}}\left(t\right) & =\left\{\begin{array}{cc} 0\; & ,\;t<t_{0}\\ g_{\text{AMPA}}\left(t_{0}\right)e^{-\left(t-t_{0}\right)/\tau_{\text{AMPA}}}\; & ,\;t\geq t_{0}\\ \; \end{array}\right. \end{array}$$

(2b) $$\begin{array}{ll} g_{\text{NMDA}}\left(t\right) & =\left\{\begin{array}{cc} 0\; & ,\;t<t_{0}\\ g_{\text{NMDA}}\left(t_{0}\right)e^{-\left(t-t_{0}\right)/\tau_{\text{NMDA}}}\; & ,\;t\geq t_{0}\\ \; \end{array}\right. \end{array}$$

(2c) $$\begin{array}{ll} g_{\text{GABA}}\left(t\right) & =\left\{\begin{array}{cc} 0\; & ,\;t<t_{0}\\ g_{\text{GABA}}\left(t_{0}\right)e^{-\left(t-t_{0}\right)/\tau_{\text{GABA}}}\; & ,\;t\geq t_{0}\\ \; \end{array}\right. \end{array}$$

Where *t* denotes the simulation time and *t*_0_ denotes the time at which an input spike is received. *g*_AMPA_ and *g*_NMDA_ represent AMPA (α-amino-3-hydroxy-5-methyl-4-isoxazolepropionic acid) and NMDA (*N*-methyl d-aspartate) receptor-mediated conductance respectively, which provide excitation, and *g*_GABA_ represents the GABA receptor-mediated conductance, which provides inhibition. *τ*_AMPA_, *τ*_NMDA_, and *τ*_GABA_ are the decaying time constants of each receptor type. The synaptic conductances were modeled as decaying exponential functions, which provide a reasonable accuracy as well as computational efficiency (Ros et al., 2006).

The parameters of each cell type (Eqs 1A–C) and synaptic receptor (Eqs 2A–C) have been chosen to model granule cell, stellate cell, and Golgi-cell dynamics (see Table 1) (Silver et al., 1996; Tia et al., 1996; Nusser et al., 1997; Rossi and Hamann, 1998).

**Table 1:** **Parameters of the model cells**.

Parameter	GrC	SC	GoC
Membrane capacitance (*C*_m_; pF)	2	4	50
Firing threshold (θ*_Vm_*; mV)	−40	−40	−50
Resting potential (*E*_rest_; mV)	−65	−56	−65
Excitatory reversal potential (*E*_AMPA_, *E*_NMDA_; mV)	0	0	0
Inhibitory reversal potential (*E*_GABA_; mV)	−65	−58	−65
Resting conductance (*G*_rest_; nS)	0.2	0.2	3
Resting time constant (τ_m_; ms)	10	20	16.7
AMPA receptor time constant (τ_AMPA_; ms)	0.5	0.64	0.5
NMDA-receptor time constant (τ_NMDA_; ms)	40	–	–
GABA-receptor time constant (τ_GABA_; ms)	10	2	10
Spikelet time constant (τ_EC_; ms)	1	1	1

*These parameters have been obtained from the following references: GrC (D’Angelo et al., 1993, 1995, 1998, 2001; Gabbiani et al., 1994; Nieus et al., 2006), SC (Häusser and Clark, 1997; Carter and Regehr, 2002; Kreitzer et al., 2002; Chavas and Marty, 2003; Suter and Jaeger, 2004), and GoC (Forti et al., 2006; Solinas et al., 2007a,b). Synaptic connectivity is defined in Table 2 and synaptic conductances are reported in Table 3*.

Table 2 shows the synaptic connections which have been included in the developed model, the convergence and divergence ratios, and the different kinds of receptors under consideration. While both AMPA and NMDA receptors have been included in MF-GrC synapses, only AMPA transmitters have been implemented at the remaining excitatory synapses (MF-GoC, GrC-GoC, and GrC-SC). This decision emerges from available evidence suggesting, through *in situ* hybridization studies, that adult GoCs do not express NR2B (Ottersen and Storm-Mathisen, 2000). Moreover, patch-clamp recording experiments suggest that NR2D-containing receptors are expressed only extra-synaptically on these cells (Cull-Candy et al., 2001; Brickley et al., 2003).

**Table 2:** **Synaptic connections in the network model**.

Connection	Divergence	Convergence	Receptors
MF-GrC	1:51.4	4:1	AMPA, NMDA
MF-GoC	1:3.9	50:1	AMPA
GoC-GrC	1:666.7	4:1	GABA
GrC-GoC	1:0.6	100:1	AMPA
GrC-SC	1:6.7	100:1	AMPA
SC-GoC	1:4.5	50:1	GABA
GoC-GoC	1:26	26:1	GABA

*This table reports the convergence and divergence ratios and the receptors implemented in the model. The GoC inhibitory connections (SC-GoC and GoC-GoC) are included only when explicitly mentioned. The convergence and divergence ratios have been taken from the literature (Nieus et al., 2006; Solinas et al., 2007a,b, 2010; Mapelli et al., 2009; Hull and Regehr, 2012)*.

### The synaptic weights

Synaptic weights have been derived from previous network models (Maex and Schutter, 1998; Solinas et al., 2010). As a strategy, we first established the weight of MF-GrC connections and then adjusted the others to calibrate network responses. Reflecting physiological determinations, the simulated network generated singlets in control and increased its output to doublets and triplets when inhibition was reduced or the MF-GrC synapse was potentiated (Mapelli and D’Angelo, 2007; Roggeri et al., 2008; Andreescu et al., 2011; Diwakar et al., 2011). We have defined three different plasticity states at the MF-GrC connection: LTD, control, and LTP (D’Errico et al., 2009). The weights in control derived from global peak conductance values (i.e., the maximum conductance resulting from the simultaneous activation of all the synapses impinging on a single GrC) measured in neurophysiological recordings. In the control state, the peak conductance of AMPA-receptor channels at each MF-GrC synapse was set at 3.49/4 = 0.87 nS (Gabbiani et al., 1994). With an AMPA/NMDA ratio of 9.94, the peak conductance of NMDA-receptor channels at each MF-GrC synapse was set at 0.87/9.94 = 0.087 nS. In the LTP (LTD) state, peak conductance at the MF-GrC connection was allowed to increase (decrease) by 30%, i.e., to 1.131 nS (0.609 nS) (Maffei et al., 2002; D’Angelo et al., 2005). The inhibitory synaptic weights at the GoC-GrC synapse were also set similar to those reported experimentally (Mapelli et al., 2009), therefore implementing an appropriate excitatory/inhibitory balance in GrCs. Once the GrC synaptic weights had been established, the model allowed investigating the full parameter space including all the synaptic weights in the inhibitory loops (that is, MF-GoC, GrC-GoC, and GoC-GrC), which ranged between the boundaries reported in Table 3. Throughout the paper, the AMPA/NMDA ratio was maintained constant and only the AMPA synaptic weight has been indicated in the figures.

**Table 3:** **Synaptic weights used for model simulations**.

Connection	LTD (nS)	Control (nS)	LTP (nS)
MF-GrC	AMPA: 0.609	AMPA: 0.87	AMPA: 1.131
	NMDA: 0.062	NMDA: 0.087	NMDA: 0.114
MF-GoC	0.5	1	2
GoC-GrC	0.75	1.5	3
GrC-GoC	1.5	3	6
GoC-GoC	0	1	3
SC-GoC	0	0.25	1

*The table reports the synaptic weights in control, LTD, and LTP states for each connection. The configurations are based on previous works (Maex and Schutter, 1998) and on preliminary simulations. The synaptic connections traditionally described in the literature (MF-GrC, MF-GoC, GoC-GrC, and GrC-GoC) have been studied conjointly (i.e., simulating all the possible four-dimensional weight combinations), while the GoC inhibitory connections (SC-GoC and GoC-GoC) have been added to simulations separately*.

It should be noted that in this paper we did not implement intrinsic learning mechanisms but rather we investigated how multiple changes at different synapses could modify the network functional state. Therefore, synaptic weights have been systematically changed either independently or in various combinations. The implementation of network learning rules for distributed synaptic plasticity goes beyond the present aims and represents a further step that will be required in order to investigate network self-organization.

### Stimulation protocol

In order to analyze the response of the network to realistic patterns, our model was stimulated with 100 Hz MF spike bursts (Mapelli and D’Angelo, 2007; D’Angelo and De Zeeuw, 2009). The individual spike times were extracted from a Gaussian distribution. In order to avoid GrC saturation, some preliminary simulations were run to study the influence of two main parameters: the firing probability of each single MF and the standard deviation of firing times. By lowering firing probability, the response of the GrCs was reduced. Similarly, by increasing sparseness of MF input activity, the amount of response decreased due to the lack of coincidence between incoming spikes. A 5-Hz basal random MF activity was included based on previous investigations (Solinas et al., 2010). In aggregate, the stimulation protocol was composed of MF bursts with a maximum of three spikes burst and average inter-spike frequency of 100 Hz. Spikes were generated with a probability of 0.7 followed a Gaussian time distribution with SD 1 ms.

### Analysis of simulated results: Excitatory/inhibitory balance

Since GrCs encode information both in the delay of the first spike and in the spike count, we have evaluated the peri-stimulus histogram (PSTH) in response to the first spike of the MF burst and the number of spikes (usually singlets, doublets, or triplets) elicited in response to the whole burst. In order to determine the activity state of neurons under combined excitatory and inhibitory synaptic drive, we have defined the GrC excitatory/inhibitory conductance balance using the following equation.

(3) $$\begin{array}{cccc} E∕Ig\;\text{balance}\left(t\right) & =\frac{1}{\#P}\underset{i\in P}{\sum }\left(\underset{j\in E_{i}}{\sum }\left(g_{\text{AMPA},i,j}\left(t\right)+g_{\text{NMDA},i,j}\left(t\right)\right)\right. & & \\ & \;\left.+\underset{j\in I_{i}}{\sum }g_{\text{GABA},i,j}\left(t\right)\right) \end{array}$$

where *E/I g* balance (*t*) represents the average excitatory/inhibitory balance at time *t*, *P* represents the set of cells under study. *E_i_* and *I_i_* represent all the sources of excitation and inhibition. *g*_AMPA*,i,j*_(*t*) and *g*_NMDA*,i,j*_(*t*) represent the AMPA-receptor and NMDA-receptor-mediated conductances at the *j-*th excitatory synapse reaching the cell *i*, as previously defined in Eq. 3. Similarly, *g*_GABA,*i,j*_(*t*) represents the GABA-receptor conductance at the *j-*th inhibitory synapses reaching the cell *i*. In Eq. 4, the excitatory conductances (AMPA and NMDA) have been considered with negative sign, and the inhibitory conductance (GABA) has been considered positive. Indeed, a negative value at the conductance balance indicates the predominance of excitation over inhibition in the cell.

## Results

In order to investigate the consequences of distributed synaptic plasticity on spike timing, we have analyzed the impact of weights at the different synapses of the granular layer in a spiking network model (Figure 1). Background activity was generated by random low-frequency activity in MFs (average frequency = 5 Hz) and three-spike 100-Hz bursts were then used to elicit network responses to impulsive stimulation.

**Figure 2:**
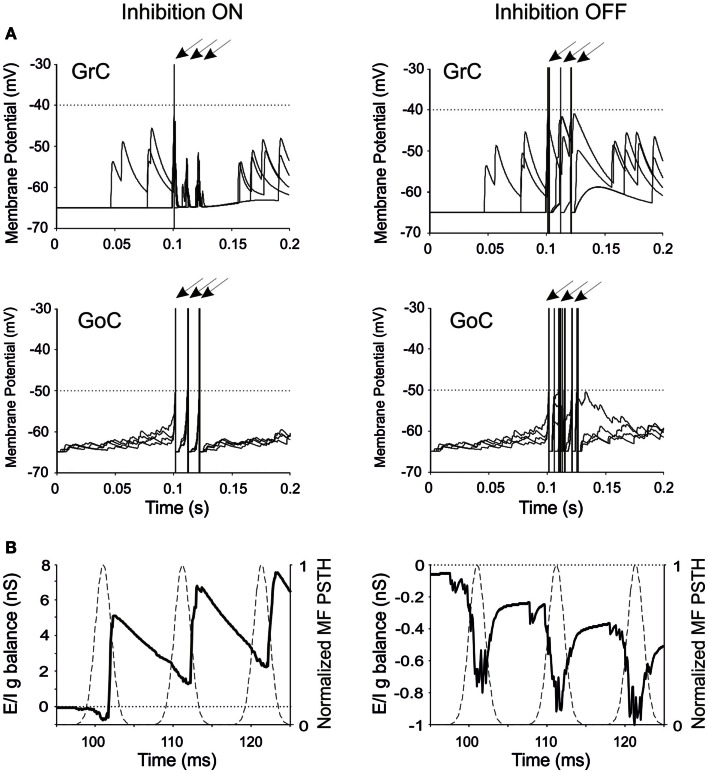
**Neuronal responses and excitatory/inhibitory conductance balance in the granular-layer model**. Response of the model to a stimulation burst composed of three spikes at 100 Hz over a 5 Hz background activity generated by random low-frequency firing in the MFs. **(A)** Electrical response of single GrCs and GoCs in control (inhibition ON) and in the absence of inhibition (inhibition OFF). In each panel, four traces are shown superimposed and arrows indicate the mossy fiber stimulation time. Note that the same seeds are used for initializing the random number generator in all simulations. The GrC and GoC output bursts are much stronger when GoC-GrC inhibition is blocked. Dotted lines represent the threshold potential for each cell model. **(B)** Average *E*/*I* *g* balance (see Methods) of the GrC population in response to the MF spike burst. Inhibitory conductance is upward, excitatory conductance is downward. In control conditions, the *E*/*I* *g* balance is in favor of excitation only in response to the fist spike, then it turns in favor of inhibition. When inhibition is turned off, the *E*/*I* *g* balance remains always negative.

The response of single neuronal elements is represented in Figure 2A. With control weight settings [see Table 3 (Solinas et al., 2010)], GrC responses manifested a marked dependence on activity in the inhibitory loops. When the inhibitory loops were active, the GrCs usually generated a single spike followed by sub-threshold membrane potential changes, while in the absence of inhibition the GrCs generated multiple spikes in response to burst stimulation (D’Angelo et al., 1995, 2001). This is an implementation of the *time-window* effect, which predicts that the feed-forward inhibitory loop passing through the MF-GoC-GrC connections can curtail the GrC response (D’Angelo and De Zeeuw, 2009). The GoCs efficiently followed the input bursts, as expected from their high reactivity to synaptic inputs through the MFs (Kanichay and Silver, 2008). GoC activity increased when GoC-GrC transmission was blocked, since GrC disinhibition enhanced activity at PF synapses. Thus, the major functional properties of the circuit revealed in electrophysiological experiments were captured by the model. Nevertheless, it must be noted that theta-frequency auto-rhythmicity of GoCs was not implemented (Forti et al., 2006).

The regulation of GrC responsiveness by inhibition was reflected by the average GrC synaptic conductance, which provided an effective measure of the Excitatory/Inhibitory balance (*E/I g balance*). Following a MF burst, the *E*/*I* *g* balance showed an initial negativity (net excitatory conductance) followed by a large positivity (net inhibitory conductance), which was eliminated by switching-off GoC-GrC transmission (Figure 2B).

**Figure 3:**
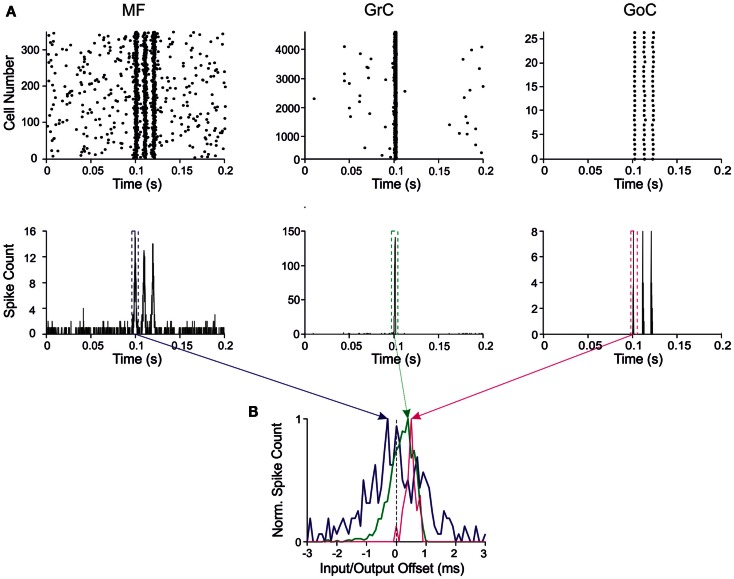
**Response patterns in the granular-layer model**. Response of the model to a stimulation burst composed of three spikes at 100 Hz over 5 Hz background activity generated by random low-frequency firing in the MFs. **(A)** Response of network elements in control conditions. (*Top*) Raster plots of activity recorded in the MF (*left*), GrC (*center*), and GoC (*right*) populations, respectively. Note the three-spike bursts in MFs with 100-Hz average frequency and firing probability 0.7. GrC activity rises sharply in response to the first spike in the burst generating a single output spike. Then GrC activity is inhibited by the GoCs, which keep on firing in response to all the three MF burst spikes. (*Bottom*) Peristimulus-time histograms (PSTH) in MFs (*left*), GrCs (*center*), and GoCs (*right*) respectively. Note the single PSTH peak in GrCs in response to the first MF burst (bin size 1 ms). **(B)** PSTHs taken from GrCs and GoCs around the first MF spike are shown over-imposed on expanded scale. The *Input/Output offset* (I/O offset) represents the time with respect to the MF stimulation. Given the probabilistic distribution of MF burst activity around a mean time value (dotted line), the I/O offset can assume negative values at the beginning of discharge. Note that the GrC and GoC PSTH peaks are delayed with respect to the MF PSTH peak due to the integration time of incoming activity in the cells.

The behavior of the entire network is represented in Figure 3A. Random low-frequency activity in MFs generated sparse GrC spikes with an average frequency of about 0.1 Hz (Solinas et al., 2010) without remarkably engaging GoCs. MF bursts raised the probability of spike emission from GrCs up to around 25%, which is in the range estimated for active granular-layer clusters activated by impulsive sensory stimulation *in vivo* (Diwakar et al., 2011). The GoCs emitted synchronous spike triplets, which caused a powerful GrC inhibition summating along the burst (Mapelli et al., 2009). In this configuration, the network efficiently filtered the second and third spikes that would otherwise be generated in the absence of inhibition. Therefore, the major question was how the weights at the various synapses could regulate and modify these filtering properties.

The input/output (I/O) function of the circuit was analyzed by evaluating the relationship between input and output bursts in GrCs, which represent the output element of the circuit. I/O plots for the first GrC spike showed the GrC response as a function of the time-offset with respect to MF discharge (Figure 3B). By counting the spikes emitted by GrCs, this PSTH summarizes the intensity, delay, and duration of the discharge and can be used to analyze the I/O behavior of the circuit.

### The effect of MF-GrC weights

**Figure 4:**
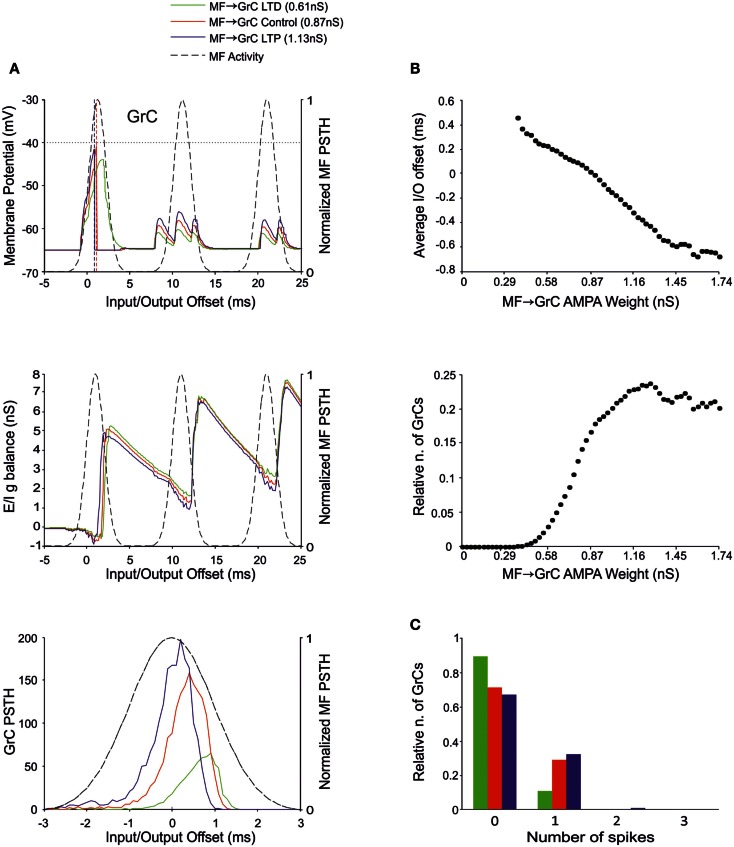
**Effect of MF-GrC synaptic weight on GrC activity**. In these simulations the following weight configuration was used: MF-GrC AMPA receptor 0–1.74 nS (NMDA-receptor weight has been proportionally set), MF-GoC 1 nS, GrC-GoC 3 nS, GoC-GrC 1.5 nS, SC-GoC 0 nS, and GoC-GoC 0 nS. **(A)** GrC response with three different MF-GrC weights (reported for AMPA): LTD (0.61 nS; green), Control (0.87 nS; red) and LTP (1.13 nS; blue). The theoretical distribution of the MF stimulation burst is shown with a dashed line. (*Top*) Membrane potential in a single GrC and its firing threshold (dotted straight line at −40 mV). (*Middle*) *E*/*I* *g* balance of the GrC population with the three MF-GrC weight settings. (*Bottom*) PSTH of the GrC response after the first spike in the burst. Note that LTP produces greater and earlier responses, while the opposite occurs with LTD. These changes in the PSTH are consistent with those in the *E*/*I* *g* balance. **(B)** Plots of the average input/output offset (*top*) and of the GrC firing probability (*bottom*) with respect to MF-GrC weights. **(C)** Relative number of GrCs generating zero, one, two, or three spikes in response to the stimulation burst.

The MF-GrC connection has been shown to express forms of LTP and LTD (Hansel et al., 2001) and has been proposed to significantly influence cerebellar signal processing (Nieus et al., 2006; D’Angelo and De Zeeuw, 2009; Arleo et al., 2010). In our simulations, synaptic weights at MF-GrC connections effectively modified spike transmission. By increasing the MF-GrC weight (LTP condition), the GrC spike response occurred earlier and with higher probability, while the opposite occurred by reducing weights (LTD condition). This caused corresponding changes in the first-spike GrC PSTH (Figure 4A), which became smaller and shifted to the right while moving from higher to lower weights. Consistently, the average I/O offset (Figure 4B) decreased monotonically by decreasing the MF-GrC synaptic weight, indicating shortening of the GrC response delay. Moreover, average GrC firing probability increased as expected from a stronger excitatory input (Figure 4B), but then tended to stabilize (or even slightly decreased) beyond a certain MF-GrC synaptic weight. The origin of this *plateau* effect is that, as the GrC response increases due to higher MF-GrC weight, the inhibitory feed-back loop is more intensely activated preventing a further increase in GrC firing (this revealed that the feed-back loop exerted a homeostatic feed-back effect, as further considered in the discussion). Finally, MF-GrC synaptic weights had an impact on the spike pattern emitted by GrCs, which changed from a small number of singlets in the LTD state toward a higher amount of singlets and some doublets in the LTP state (Figure 4C). Further modulation of this pattern will occur by combining plastic changes at multiple synaptic sites, as reported below.

### The effect of MF-GoC weights

**Figure 5:**
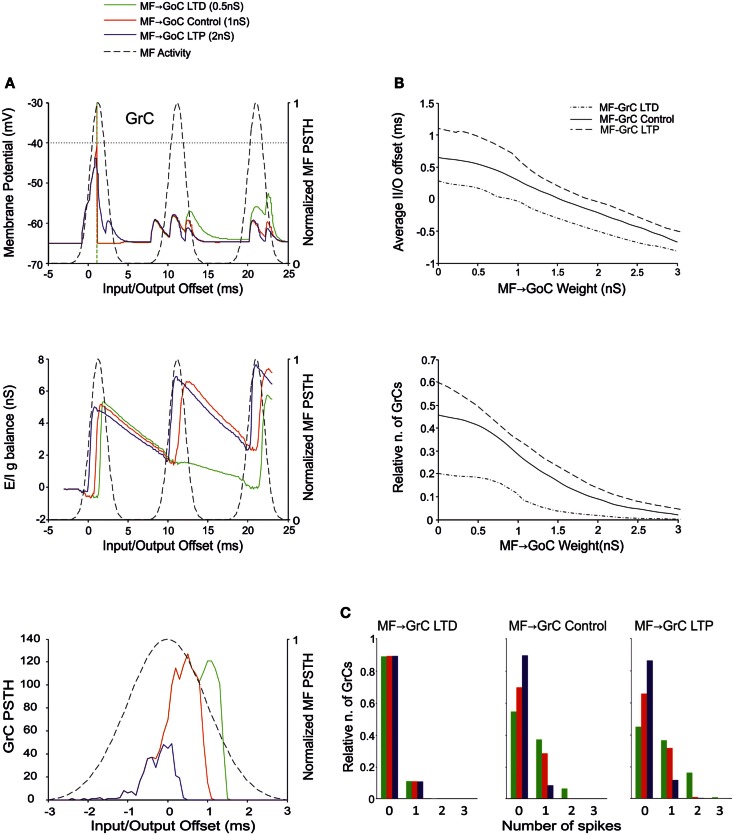
**Effect of MF-GoC synaptic weight on GrC activity**. In these simulations the following weight configuration was used: MF-GrC 0.61 nS (LTD), 0.87 nS (control), or 1.87 nS (LTP) (AMPA-receptor weight), MF-GoC 0–3 nS, GrC-GoC 3 nS, GoC-GrC 1.5 nS, SC-GoC 0 nS, GoC-GoC 0 nS. **(A)** GrC response with MF-GrC control configuration and three different MF-GoC weights: LTD (0.5 nS, green), Control (1 nS, red), and LTP (2 nS, blue). The theoretical distribution of the MF stimulation burst is shown with a dashed line. (*Top*) Membrane potential in a single GrC and its firing threshold (dotted straight line at −40 mV). (*Middle*) *E*/*I* *g* balance of the GrC population. (*Bottom*) PSTH of the GrC response after the first spike in the burst. MF-GoC LTD produces greater and more protracted responses, while the opposite occurs with LTP. **(B)** Average input/output offset (*top*) and GrC firing probability (*bottom*) with respect to MF-GoC weights. The changes observed in conjunction with three different MF-GrC weights are shown for comparison (MF-GrC LTD dot dash line; MF-GrC control solid line; MF-GrC LTP dashed line). **(C)** Relative number of GrCs generating zero, one, two, or three spikes in response to the stimulation burst. These are reported for three different MF-GrC weights, as specified in **(B)**.

There is currently no evidence for long-term synaptic plasticity between MFs and GoCs, but its potential existence is of relevance. Here we considered that, in the model, a change in MF-GoC weights could have as much the same effect as a change in the number of active MF-GoC synapses, which is a plausible mechanism of GoC regulation. By adjusting the MF-GoC synaptic weights, the model showed remarkable changes in GrC activation (Figure 5A). Not unexpectedly, higher weights at the GoC input anticipated GoC firing and GoC inhibition on the GrCs. The final effect was to reduce the time window for GrC firing and therefore the GrC firing probability. This is clearly reflected into the GrC PSTH, which shows a shortening of the time window as the MF-GoC weights move from LTD to LTP. In parallel the GrC *E*/*I* *g* balance showed an increase in inhibitory strength (Figure 5B). The impact of MF-GoC synaptic weights on the spike pattern emitted from GrCs was to change it from low number of singlets in the LTP state toward higher number of singlets and a considerable amount of doublets in the LTD state (Figure 5C). These effects were modulated by plasticity at the MF-GrC synapse, in a way that the amount of doublets markedly increased with combined increase of MF-GrC and decrease of MF-GoC weights. These observations suggest that distribution of plasticity at multiple sites can effectively regulate the GrC firing pattern, as further considered below.

### The effect of GoC-GrC weights

**Figure 6:**
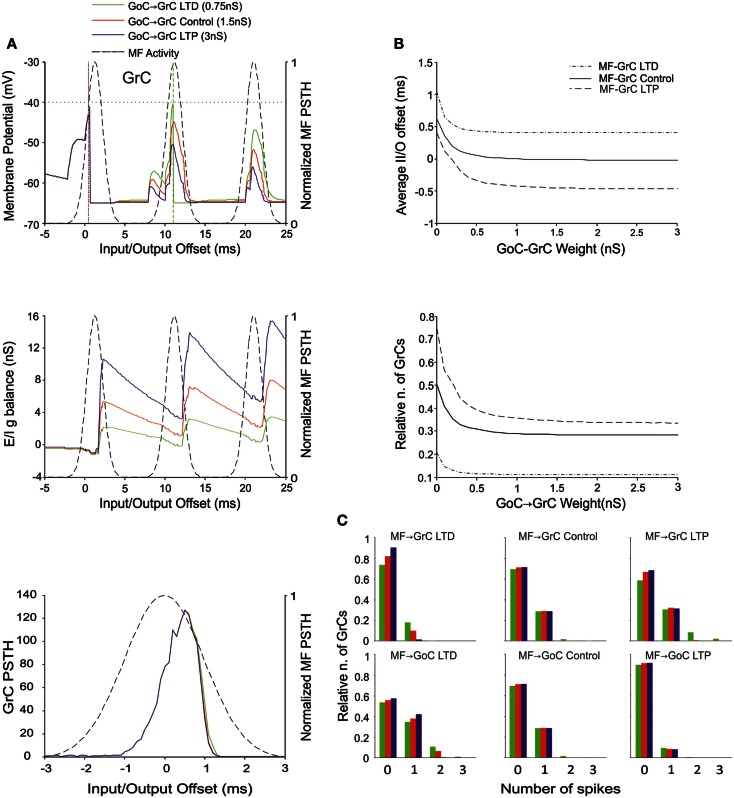
**Effect of GoC-GrC synaptic weight on GrC activity**. In these simulations the following weight configuration was used: MF-GrC 0.61 nS (LTD), 0.87 nS (control), or 1.13 nS (LTP) (AMPA-receptor weight), MF-GoC LTD (0.5 nS), Control (1 nS) and LTP (2 nS), GrC-GoC 3 nS, GoC-GrC 0-3 nS, SC-GoC 0 nS, GoC-GoC 0 nS. **(A)** GrC response with MF-GrC and MF-GoC control configurations and three different GoC-GrC weights: LTD (0.75 nS, green), Control (1.5 nS, red), and LTP (3 nS, blue). The theoretical distribution of the MF stimulation burst is shown with a dashed line. (*Top*) Membrane potential in a single GrC and its firing threshold (dotted straight line at −40 mV). (*Middle*) *E*/*I* *g* balance of the GrC population. (*Bottom*) PSTH of the GrC response after the first spike in the burst. GoC-GrC did not influence the GrC response during the initial part of the burst. **(B)** Average input/output offset (*top*) and GrC firing probability (*bottom*) with respect to GoC-GrC weights. The changes observed in conjunction with three different MF-GrC weights (LTD dot dash line; control solid line; LTP dashed line) are shown for comparison. **(C)** Relative number of GrCs generating zero, one, two, or three spikes in response to the stimulation burst. These are reported for three different MF-GrC weights and for three MF-GoC weights. GoC-GrC LTD weight noticeably increased the proportion of GrCs firing doublets and even triplets (especially with MF-GrC LTP and with MF-GoC LTD].

There is currently no evidence for LTP or LTD at the GoC-GrC synapse, although protracted forms of regulation with a potential homeostatic significance have been reported (Rossi et al., 2006; Mapelli et al., 2009; Brandalise et al., 2012). Our simulations showed that higher weights reduced GrC firing and lower weights enhanced GrC firing, but these changes were poorly effective in controlling the initiation of GrC activity. Thus, the GrC PSTH showed no remarkable changes concerning the first-spike probability and timing (Figure 6A). Nonetheless, the GrC I/O *g* balance showed remarkable changes after emission of the first spike, bringing about remarkable consequences for the generation of subsequent spikes. Accordingly, no remarkable changes were observed in I/O offset and firing probability plots for the first GrC spike (Figure 6B). The impact of GoC-GrC synaptic weights on the spike pattern emitted from GrCs was to change it from singlets in the high-weight state toward doublets and triplets in the low-weight state (Figure 6C). These effects were modulated by plasticity at other synapses in the feed-forward loop, in a way that the amount of doublets and triplets markedly increased when a decrease in GoC-GrC weights was combined with an increase in MF-GrC weights and a decrease in MF-GoC weights.

### The effect of GrC-GoC weights

**Figure 7:**
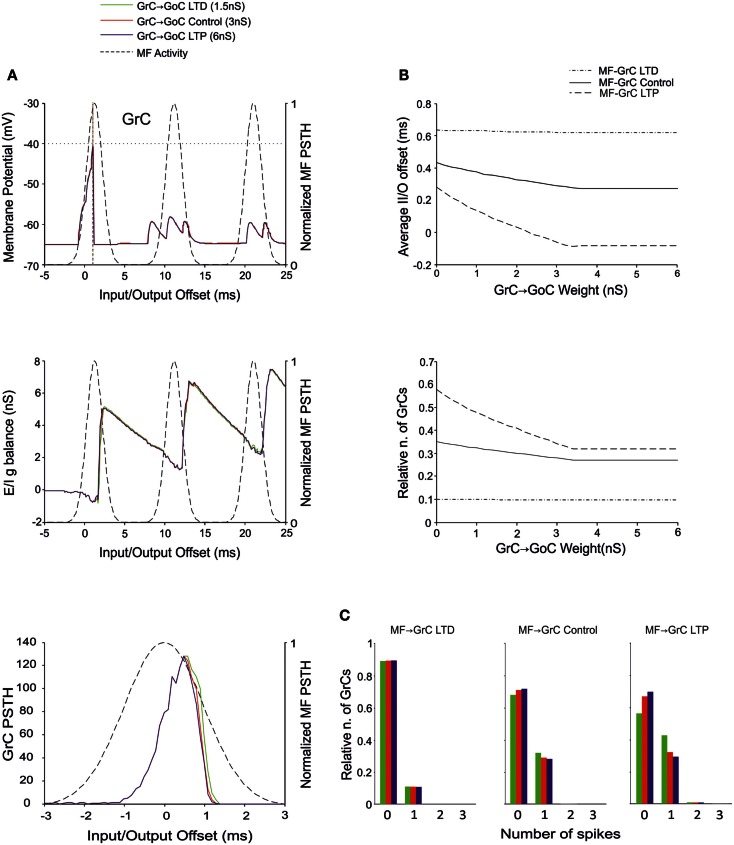
**Effect of GrC-GoC synaptic weight on GrC activity**. In these simulations the following weight configuration was used: MF-GrC 0.61 nS (LTD), 0.87 nS (control), or 1.13 nS (LTP) (AMPA-receptor weights), MF-GoC 1 nS, GrC-GoC 3 nS, GoC-GrC 1.5 nS, SC-GoC 0 nS, GoC-GoC 0 nS. **(A)** GrC response with MF-GrC control configuration and three different GrC-GoC weights: LTD (1.5 nS, green), Control (3 nS, red), and LTP (6 nS, blue). The theoretical distribution of the MF stimulation burst is shown with a dashed line. (*Top*) Membrane potential in a single GrC and its firing threshold (dotted straight line at −40 mV). (*Middle*) *E*/*I* *g* balance of the GrC population. (*Bottom*) PSTH of the GrC response after the first spike in the burst. GrC-GoC did not influence the GrC response during the initial part of the burst in MF-GrC control conditions. **(B)** Average input/output offset (*top*) and GrC firing probability (*bottom*) with respect to GrC-GoC weights. The changes observed in conjunction with three different MF-GrC weights (LTD dot dash line; control solid line; LTP dashed line) are shown for comparison. Note that the effect of GrC-GoC becomes patent only in conjunction with medium/high MF-GrC weights (control and especially LTP). **(C)** Relative number of GrCs generating zero, one, two, or three spikes in response to the stimulation burst. These are reported for three different MF-GrC configurations.

Recently, the existence of forms of plasticity at the GrC–GoC connection has been reported suggesting that this synapse can undergo persistent transmission changes (Robberechts et al., 2010). Our simulations showed that higher weights reduced GrC firing and lower weights enhanced GrC firing, but these changes were poorly effective in controlling the initiation of GrC activity. Indeed, this inhibition reached the GrCs only at the final part of the excitatory window due to accumulation of delays in the feed-back inhibitory loop. Thus, the GrC PSTH showed no remarkable changes concerning the first-spike probability and timing (Figure 7A). Indeed, the GrC conductance balance showed no remarkable differences compared to control (cf. also Figure 4A). The inhibitory strength was similar with low GoC-GrC weights and with high GoC-GrC weights. Accordingly, no remarkable changes were observed in I/O offset and firing probability plots for the first GrC spike, unless when the MF-GrC weight were set at medium-high values (Figure 7B). Nonetheless, the impact of GrC-GoC synaptic weights was to control the emission of spikes when the activity of the GrC was high (Figure 7C). Thus, the combination of high weights at MF-GrC and GrC-GoC connections effectively shifted the average firing time in the GrC while keeping the firing rate at the same range of activity. These results show that the feed-back loop effectively behaves as a homeostatic mechanism of the granular activity.

### The effect of inhibition onto GoCs

**Figure 8:**
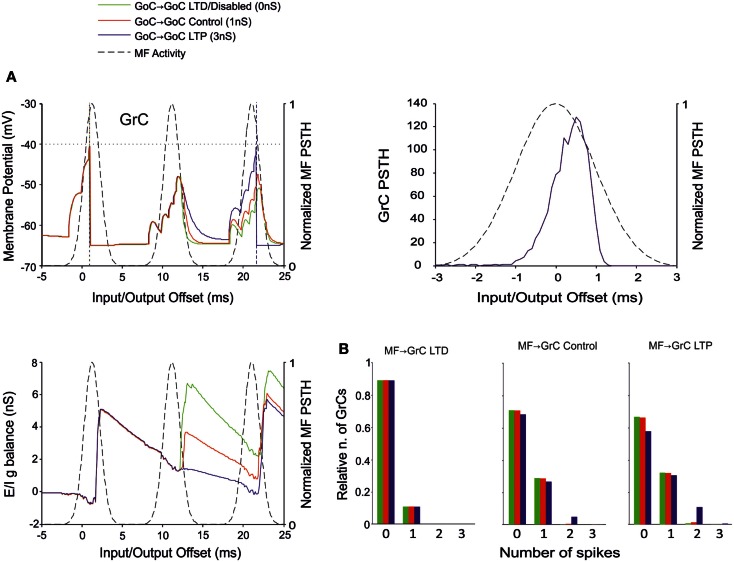
**Effect of GoC-GoC synaptic weight on GrC activity**. In these simulations the following weight configuration was used: MF-GrC 0.61 nS (LTD), 0.87 nS (control), or 1.13 nS (LTP) (AMPA-receptor weight), MF-GoC 1 nS, GrC-GoC 3 nS, GoC-GrC 1.5 nS, SC-GoC 0 nS, GoC-GoC 0 nS (LTD/disabled, green), 1 nS (control, red), or 3 nS (LTP, blue). **(A)** GrC response with MF-GrC control configuration and the three GoC-GoC configurations. The theoretical distribution of the MF stimulation burst is shown with a dashed line. (*Top left corner*) Membrane potential in a single GrC and its firing threshold (dotted straight line at −40 mV). (*Bottom Left*) *E*/*I* *g* balance of the GrC population. (*Top right corner*) PSTH of the GrC response after the first spike in the burst. GoC-GoC did not influence the GrC responsiveness during the initial part of the burst. **(B)** Relative number of GrCs generating zero, one, two, or three spikes in response to the stimulation burst. These are reported for three different MF-GrC configurations. Note that GoC-GoC LTP configuration noticeably increased the proportion of GrCs that fire doublets in MF-GrC control and LTP configurations.

**Figure 9:**
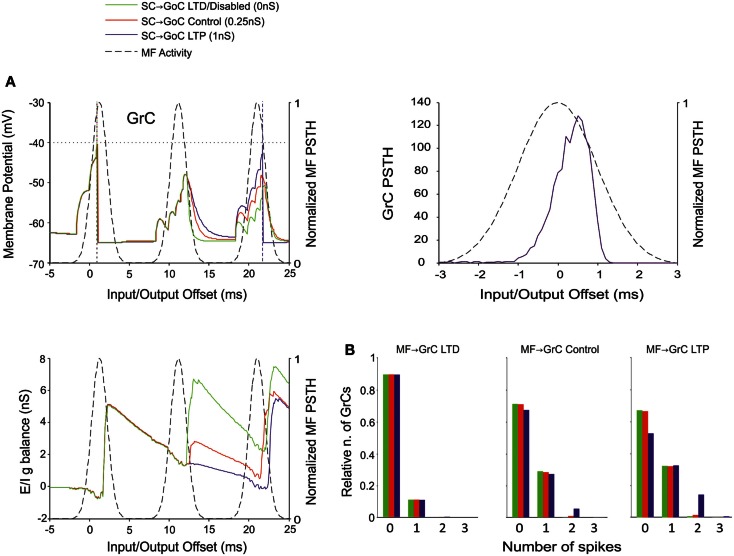
**Effect of SC-GoC synaptic weight on GrC activity**. In these simulations the following weight configuration was used: MF-GrC 0.61 nS (LTD), 0.87 nS (control), or 1.13 nS (LTP) (AMPA-receptor weight), MF-GoC 1 nS, GrC-GoC 3 nS, GoC-GrC 1.5 nS, SC-GoC 0 nS (LTD/disabled, green), 0.25 nS (control, red), 1 nS (LTD, blue), GoC-GoC 0 nS. **(A)** GrC response with MF-GrC control configuration and three different SC-GoC configurations. The theoretical distribution of the MF stimulation burst is shown with a dashed line. (*Top left corner*) Membrane potential in a single GrC and its firing threshold (dotted straight line at −40 mV). (*Bottom Left*) *E*/*I* *g* balance of the GrC population. (*Top right corner*) PSTH of the GrC response after the first spike in the burst. SC-GoC did not influence the GrC responsiveness during the initial part of the burst in MF-GrC control conditions. **(B)** Relative number of GrCs generating zero, one, two, or three spikes in response to the stimulation burst. These are reported for three different MF-GrC configurations. Similarly to the GoC-GoC connection reported in Figure 8, SC-GoC LTP configuration noticeably enhanced the proportion of GrCs that fire doublets in MF-GrC control and LTP.

Recent results have provided evidence for two modalities of GoC inhibition, namely through GoC-GoC (Hull and Regehr, 2012) and SC-GoC (Casado et al., 2000) connections. In this model, both the GoC-GoC and SC-GoC synapses proved able to regulate the generation of GrC spikes in response to MF burst stimulation. With either GoC-GoC or SC-GoC synaptic connections, the GoCs tend to generate action potentials every second spike of the MF burst, while keeping the GrCs at a reduced level of inhibition in correspondence of other spikes (Figures 8A and 9A). Thus, higher GoC-GoC or SC-GoC weights enhanced GrC firing, while lower weight reduced GrC firing without affecting the initiation of GrC activity. Consistently, the GrC PSTH showed no remarkable changes concerning first-spike probability and timing (Figures 8A and 9A). Nonetheless, in the I/O GrC conductance balance, the inhibitory strength for subsequent spikes was reduced with high GoC-GoC weights and increased with low GoC-GoC weights (Figure 8A). Accordingly, no remarkable changes were observed in I/O offset and firing probability plots for the first GrC spike (figure not shown), but the impact of GoC-GoC synaptic weights was observed on the emission of late spikes. Eventually, the GoC-GoC connection could markedly increase the emission of doublets and even some triplets in the high-weight state (Figure 8B).

A similar behavior was observed by regulating the weights of SC-GoC loop (Figure 9), although we could not take into account the complex regulatory mechanisms of the molecular layer interneuron network, which could substantially modify the impact of this pathway.

## Discussion

This paper shows that *distributed synaptic plasticity* allows simultaneously regulating multiple processing features of the cerebellum granular-layer network. By adjusting synaptic weights at the MF-GrC synapse, in feed-back and feed-forward inhibitory loops and in the interneuron inhibitory network, the probability, positioning, and number of spikes emitted by the GrCs changed generating quasi-digital spike patterns. The relevance of these effects for cerebellar regulatory mechanisms and network computation is discussed.

### Control of spike timing by distributed synaptic plasticity

Simulations showed that MF bursts caused the emission of GrC spikes through a permissive time-window limited by inhibition, which controlled the evolution of the response (D’Angelo and De Zeeuw, 2009). The precise timing of the *first spike* was mostly regulated by the MF-GrC connection strength. Then, the granular-layer inhibitory loops regulated GrC activity in response to the second and subsequent spikes in the MF burst. In particular, simulations showed that LTP at MF-GrC synapse reduced the GrC reaction times and LTP at MF-GoC shortened the excitatory time-window generating more precise (i.e., less time-dispersed) PF responses. The synaptic weights at GoC-GrC connections influenced the strength and duration of the inhibitory window. In the feed-back inhibitory loop, regulation of weights at the GrC-GoC synapses transformed the increasing activity at the PFs in shorter, and more precise excitatory windows. The inhibitory connections impinging onto GoCs effectively controlled generation of *late spikes*. The control of synaptic weights in the feed-forward and feed-back inhibitory loops and in the interneuron inhibitory network indeed allowed generating a large variety of patterns in GrCs determining the number and timing of emitted spikes. While long-term synaptic plasticity has been demonstrated at the MF-GrC synapse LTD may indeed occur at the PF – GoC synapse (Robberechts et al., 2010), it is currently unknown if and how the MF-GoC, GoC-GrC, SC-GoC, and GoC-GoC connections undergo plastic changes, whose investigation is therefore of interest. In general, the concept of plasticity should refer to any kind of changes in the number and strength of connections between these neurons occurring during ontogenesis or as a consequence of modulation and learning.

The present model was made of LIF neurons and was not endowed with realistic ionic channels properties of the kind characterizing neuronal membranes and synaptic connections. Therefore, it revealed fundamental network-dependent properties, which could then be compared with intrinsic properties of neurons and synapses. Interestingly, the most relevant properties of neurons and synapses reported so far are congruent with the network properties reported here. (i) The nature of GrC synaptic receptors is such that they can precisely control both first-spike timing on the sub-millisecond scale through AMPA receptors (Silver et al., 1992; D’Angelo et al., 1995; Cathala et al., 2003) and the continuation of burst discharge through NMDA-receptors (D’Angelo et al., 1990). (ii) Presynaptic regulation of release probability during LTP and LTD (Nieus et al., 2006; D’Errico et al., 2009) efficiently regulates first-spike timing. (iii) GrC and GoC discharge properties revealed by electrophysiological investigations (D’Angelo et al., 1998; Forti et al., 2006; Solinas et al., 2007b; Kanichay and Silver, 2008) are consistent with network behaviors emerging in our experiments. In aggregate, the cellular and synaptic properties of the granular-layer match fundamental regulatory properties embedded into the network structure and tuned by distributed synaptic plasticity.

### The effect of combining synaptic changes at multiple sites

**Figure 10:**
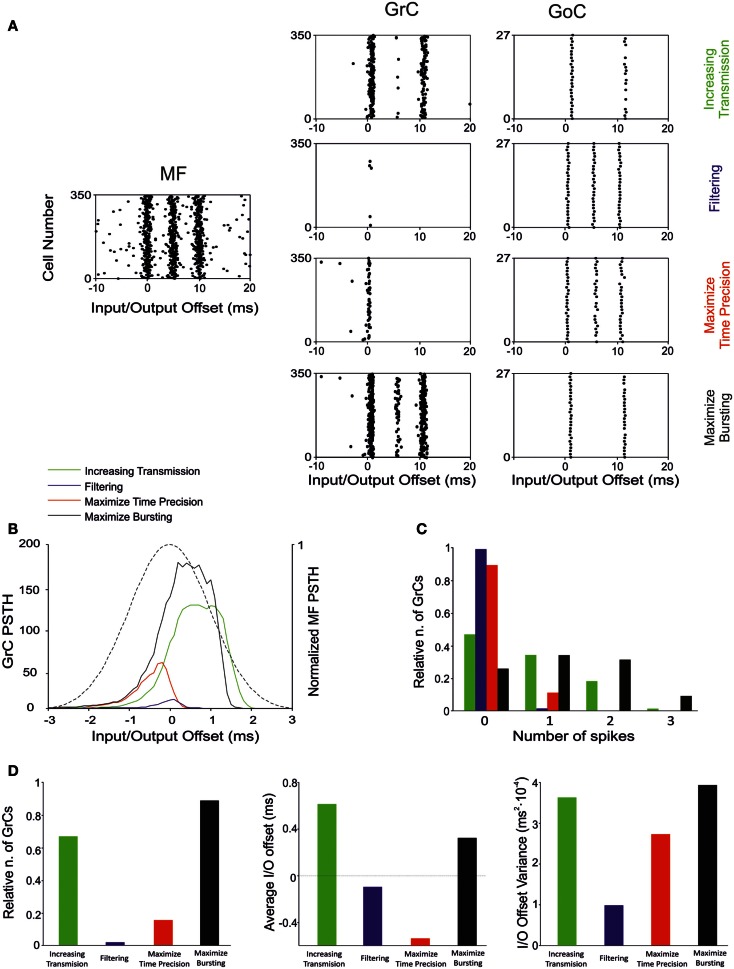
**Effect of suggested weight configurations in the GrC response**. In these simulations the weights were set according to the four configurations reported in Table 4: increasing transmission (green), filtering (blue), maximize time precision (red), and maximize bursting (black). **(A)** Raster plots of the network responses to the same MF stimulation (*left*) with each weight configuration. Raster plots of activity recorded in the GrC (*center*) and GoC (*right*) populations with the hypothesized weight configurations (one per row), respectively. **(B)** PSTH of the GrC response after the first spike in the burst. **(C)** Relative number of GrCs generating zero, one, two, or three spikes in response to the stimulation burst. **(D)** (*Left*) Relative number of GrCs generating one spike after the first spike in the stimulation burst. Average (*center*) and offset variance (*right*) of the spikes elicited by the GrCs in response to the first spike in the burst. *Maximize time precision* configuration noticeably reduces and anticipate the activity of the GrCs. *Increasing transmission* configuration enhances the activity in response to the first and second spikes in the burst. *Maximize bursting* configuration increases the number of doublets and triplets elicited. Finally, *filtering* configuration nearly avoided the GrCs firing.

The present simulations show that there are combinations of synaptic weight changes, which can achieve differential control over network processing, alternatively increasing or filtering spike transmission, maximizing first-spike precision or bursting (Table 4; Figure 10).

**Table 4:** **Summary table of combinations of synaptic plasticity changes**.

Synapses	Increasing transmission	Filtering	Maximize time precision	Maximize bursting
MF-GrC	Control	LTD	LTP	LTP
MF-GoC	LTD	LTP	LTP	LTD
GrC-GoC	LTD	Not relevant	LTP	LTD
GoC-GrC	LTD	LTP	LTP	LTD
GoC-GoC	LTP	Not relevant	LTP	LTP
SC-GoC	LTP	Not relevant	LTP	LTP
Description	The number of spikes transmitted through the MF-GrC relay is maximized at the expense of spike timing precision and noise filtering	The GrCs remain predominantly silent by strengthening all inhibitory loops. This mechanism allows filtering uncorrelated incoming activity	The precision of spike emission through the MF-GrC relay is maximized by raising the probability of the first spike and reducing late spikes in the burst	The GrCs emit doublets or triplets that could regulate Purkinje cell activity

*The main transmission pathway is indicated in red, the feed-back and feed-forward GrC inhibitory loops are indicated in blue, and the GoC inhibitory loops are indicated in green. The specific synaptic weights, which define either LTD or control or LTP states in every single synapsis, are shown in Table 3*.

#### Increasing transmission

Spike transmission through the MF-GrC relay can be increased by lowering activity in the feed-forward and feed-back inhibitory loops and by strengthening inhibition of GoCs. This occurs at the expense of first-spike precision and bursting. It is not clear when this condition could be exploited, as there is normally a strong inhibitory activity in the granular layer (Roggeri et al., 2008). However, there could be activity states in which maximizing transmission might be useful to improve subsequent pattern emergence through learning. Indeed, LTP generation is strongly dependent on removal of inhibition, which could be controlled by local release of specific neuromodulators (Prestori et al., 2013). Plasticity would then turn network weight settings in favor of first-spike precision or bursting.

#### Filtering

Filtering requires LTD at the MF-GrC synapse and high activity in the feed-forward inhibitory loop. LTD at the MF-GrC synapse may reflect protracted uncorrelated low-frequency activity in MFs. In this state, the network can filter spurious spikes allowing transmission of just highly synchronous spikes. Interestingly, various mechanisms based on NMDA and GABA-A receptors, make this filtering function of the granular-layer frequency-dependent (Mapelli et al., 2010).

#### Maximize time precision

The precision of first-spike emission can be maximized by raising all the weights. This state may emerge at the end of a learning process and is likely to be modified in accordance with the needing for spike filtering and patterning. Traditional pattern recognition models are based on fast responses to known patterns and on the absence of responses to non-correlated activity (Masquelier et al., 2008). Accordingly, the GrCs fire quickly in response to the earliest spikes of the MF burst and the inhibitory loops silence the GrC response to the latest spikes of such burst. The GoC inhibitory connections maintain a high GrC precision by anticipating the onset of inhibition and preventing multiple GrC spikes. The plausibility of this mechanism is evident when considering the numerous mechanisms implementing high-precision first-spike timing in GrCs (Silver et al., 1992; Cathala et al., 2003). Moreover, the information transmitted through the MF-GrC relay is largely due (about 50%) to millisecond precision first-spike timing (Arleo et al., 2010).

#### Maximize bursting

The bursts emitted by GrCs, which are composed of spike doublets/triples, in response to MF input bursts, can be optimized by combining LTP at the MF-GRC synapse with low activity in GoCs (obtained by weakening the inhibitory loops and by strengthening GoC inhibition). The composition of the output burst is critical for cerebellar network computation, as shown by the powerful regulation exerted by NMDA receptors (D’Angelo et al., 1995) and by the motor dysfunction that emerges when this control system is disrupted (Andreescu et al., 2011). The generation of doublets/triples could be critical to control activation of Purkinje cells (PCs) and molecular layer interneurons, which are highly sensitive to temporal summation through short-term synaptic plasticity (Dittman et al., 2000) and may also control long-term synaptic plasticity at the same synapses (Casado et al., 2000).

As long as spike bursts are regulated to achieve specific computational effects, the granular-layer circuit needs to maintain a homeostatic balance in order to prevent saturation of PF activity (Marr, 1969). The granular-layer circuit is intrinsically homeostatic in that GrC activity can be depressed by raising activity in the feed-back inhibitory loop. It is probable that homeostasis occurs, together with the various optimization processes, in order to balance network activity. Homeostasis may extend over space, for example balancing LTP and LTD over neighboring granular-layer areas (Mapelli and D’Angelo, 2007; Diwakar et al., 2011). There is also evidence that homeostasis may exploit specific mechanisms raising GrC responsiveness when inhibition is persistently increased (Rossi et al., 2006; Mapelli et al., 2009; Brandalise et al., 2012). Finally, it should be noted that during protracted bursts GrC firing is maintained higher than expected from a pure time-window mechanism (Kanichay and Silver, 2008; Mapelli et al., 2010), suggesting that additional mechanisms are indeed at work. Further physiological experiments and larger scale models should be developed to investigate the issue.

### Millisecond-precise quasi-digital spike pattern

By fully implementing the *time-window* mechanism (D’Angelo and De Zeeuw, 2009; Solinas et al., 2010), distributed synaptic plasticity can fine-tune the initiation of first-spike emission as well as the burst spike pattern (Figure 10). Regulation of first-spike delay is almost fully determined at the MF-GrC synapse with millisecond precision. Low MF-GrC weights and high activity in the inhibitory loops favor generation of singlets, while high MF-GrC weights and low activity in the inhibitory loops favors generation of doublets and triplets. The exact number of spikes emitted in specific functional contexts is not fully clear. In response to a single MF impulse, local field potentials, cell-attached and whole-cell recordings in brain slices reveal mostly singlets, while doublets and triplets become common after blocking synaptic inhibition and generating MF-GrC LTP (Mapelli and D’Angelo, 2007; Andreescu et al., 2011). In anesthetized rats *in vivo*, sensory stimulation generates short spike bursts and whole-cell recordings from GrCs show generation of a new burst (Chadderton et al., 2004; Rancz and Hausser, 2006; Duguid et al., 2012). Spike patterns reported *in vivo* in response to MF bursts show on average five EPSCs and one to three spikes in GrCs (e.g., see Duguid et al., 2012), and the composition of GrC bursts is regulated by the inhibitory circuit. In these papers, emphasis has been put on tonic inhibition (a form of inhibition caused by ambient GABA in the cerebellar glomerulus), but the impact of the inhibitory circuit on spike timing or the duration of the discharge has not been analyzed. However, in local field potential recordings *in vivo*, an apparent reduction of response duration was observed when the inhibitory circuit was blocked and a clear anticipation of the response emerged when LTP was induced at the MF-GrC synapse (Roggeri et al., 2008; Diwakar et al., 2011), as much as it was observed in the PSTHs reported in this paper (see Figures 4, 5, and 10). Therefore, the onset and duration of spike bursts in GrCs can be regulated *in vivo* in a way consistent with that predicted here.

The importance of this quasi-digital GrC spike pattern becomes evident when considering that PC responses are differentially sensitive to the number of spikes transmitted by GrCs along the PFs. First of all, the PF-PC synapse shows a pronounced short-term facilitation, so that single PF spikes are not transmitted but transmission becomes effective with two or more spikes (Casado et al., 2000; Dittman et al., 2000). A more puzzling effect is that presynaptic LTD at the PF-PC synapse is induced by spike triplets, which are needed to unblock presynaptic NMDA receptors (Casado et al., 2000). Importantly, a precise timing is implied by the millisecond precision of PC responses in relation to movements (Timmann et al., 1999; Osborne et al., 2007).

### General conclusions and implications for cerebellar network computation

These simulations show that distributed synaptic plasticity fully implements the *time-window* mechanism (D’Angelo and De Zeeuw, 2009) causing the emission of quasi-digital spike patterns, with differential regulation of the precision and probability of the first spike compared to that of late spikes. This, in turn, gives a specific significance to distributed plasticity, which is shown to control spike transmission much better than plasticity at a single synapse. Moreover, distributed synaptic plasticity can determine multiple activity states of the network, alternatively increasing or filtering transmitted spikes or maximizing first-spike precision and bursting. These states may be inter-converted, modified, or stabilized by exploiting biological properties of plasticity, like reversibility and consolidation. In this flexible scenario, the specific asset of synaptic weights at a given time could be strictly dependent on ontogenetic factors and on gating processes controlling network plasticity in relation to brain states (Schweighofer et al., 2004). Most of the information available on the potential mechanisms controlling the formation of plasticity in the cerebellar granular-layer network concerns the MF-GrC relay. For example, acetylcholine (Prestori et al., 2013) can gate MF-GrC LTP raising precision and bursting of GrC spiking. In response to MF bursts, nitric oxide (NO) can favor MF-GrC LTP and orchestrate the LTP/LTD balance in the surrounding circuit area (Maffei et al., 2003). A powerful organizing mechanism for LTP and LTD could also be provided by theta-frequency oscillation and resonance (Gandolfi et al., 2013). Finally, mechanisms intrinsic to the cerebellar glomerulus can raise GrC excitability under conditions of weak MF transmission or strong GoC activity (Mitchell and Silver, 2003; Rossi et al., 2006; Brandalise et al., 2012) and other mechanisms located on GoC dendrites can silence the Golgi cell following intense GrC – GoC transmission (Watanabe and Nakanishi, 2003). This latter set of synaptic and non-synaptic mechanisms could implement a homeostatic balance preventing neuronal activity from exceeding the functional limits of the network. The identification of the biochemical and physiological mechanisms orchestrating this multitude of network operations and determining network learning represents a challenge for future research.

These simulations also address one major issue in cerebellar modeling. Theoretical models of the cerebellum assume that learning is driven by some optimization factors related to gain, signal-to-noise ratio, and mutual information transfer. Although some models consider only learning at the PF-PC synapse (e.g., Schweighofer et al., 1998; Medina and Mauk, 2000; Kawato et al., 2011), others implement learning (Schweighofer et al., 2001) or non-recurrent state-generation in the granular layer (Yamazaki and Tanaka, 2007). Here we show that timing and bursting could also be important parameters, which can be optimized by distributed plasticity in the granular layer. Future theoretical modeling should consider this issue.

## Conflict of Interest Statement

The authors declare that the research was conducted in the absence of any commercial or financial relationships that could be construed as a potential conflict of interest.
